# International consensus to define outcomes for trials of chemoradiotherapy for anal cancer (CORMAC-2): defining the outcomes from the CORMAC core outcome set

**DOI:** 10.1016/j.eclinm.2024.102939

**Published:** 2024-12-05

**Authors:** Robert Samuel, Stephen R. Knight, Richard Adams, Prajnan Das, Jennifer Dorth, David Finch, Marianne G. Guren, Maria A. Hawkins, Susan Moug, Lakshmi Rajdev, David Sebag-Montefiore, Andrew G. Renehan, Rebecca Fish, plus the CORMAC-2 collaborators, Miguel A. Rodriguez-Bigas, Pratik Adusumilli, Ahmed Allam Mohamed, Mario Alvarez Gallego, Eva Angenete, Ane Appelt, Maaike Berbee, Danielle Brogden, Peter Brown, Lucy Buckley, Nathalie Casanova, Rachel Cooper, Nuno Couto, Peter Coyne, Tamzin Cuming, Charlotte Deijin, Kristopher Dennis, Cathy Eng, Alexandra Gilbert, Duncan Gilbert, Karyn Goodman, Rashmi Jadon, Anders Johnsson, Arunansu Kar, Ethan Ludmir, Marie-Louise Lydrup, Ivan Lyra-Gonzalez, Stefania Manfrida, Rebecca Muirhead, Sarah O'Dwyer, Thomas Rackley, Lukasz Raszewski, Leslie Samuel, Mark Saunders, Andrew Scarsbrook, Eva Segelov, Timothy Simmons, Paul Sutton, Nicholas Symons, Deborah Williamson

**Affiliations:** a NIHR Manchester Biomedical Research Centre, Division of Cancer Sciences, School of Medical Sciences, Faculty of Biology Medicine and Health, University of Manchester, Manchester, UK; b University of Leeds, Leeds Institute for Medical Research, St. James's University Hospital, Leeds, UK; c Centre for Medical Informatics, Usher Institute, University of Edinburgh, Edinburgh, UK; d School of Health and Wellbeing, University of Glasgow, Glasgow, UK; e Centre for Trials Research, Cardiff University School of Medicine, Cardiff, UK; f University of Texas MD Anderson Cancer Center, USA; g Department of Radiation Oncology, University Hospitals Cleveland Medical Centre, Cleveland, USA; h Colorectal and Peritoneal Oncology Centre, The Christie NHS FT, Manchester; i Department of Oncology, Oslo University Hospital, Institute of Clinical Medicine, University of Oslo, Oslo, Norway; j Department of Medical Physics and Biomedical Engineering, University College London, London, UK; k Department of Colorectal Surgery, Royal Alexandra Hospital, Paisley, UK; l Tisch Cancer Institute, Icahn School of Medicine at Mount Sinai, New York, NY, USA

**Keywords:** Core outcome set, Outcomes, Anal cancer, Consensus, Delphi, Chemoradiotherapy

## Abstract

Variation in outcomes definitions and reporting limit the utility of clinical trial results. The Core Outcome Research Measures in Anal Cancer (CORMAC) project developed a core outcome set (COS) for chemoradiotherapy trials for anal squamous cell carcinoma (ASCC) through an international healthcare professional and patient consensus process. The CORMAC-COS comprises 19 outcomes across 4 domains (disease activity, survival, toxicity, life impact). In CORMAC-2 we have established standardised definitions for the 11 disease activity and survival outcomes in the CORMAC COS. Definitions were agreed through a 3 step process, initially identifying existing definitions through systematic review (registered with PROSPERO, CRD42016036540), using these to populate a two-round Delphi questionnaire completed by 51 experts from 13 countries, and finally ratification through an online consensus meeting. Standardising the definitions for these core outcomes facilitates real world utilisation of the CORMAC-COS, thereby increasing the quality of data available for clinical decision-making and ultimately enhancing patient care.

## Introduction

The incidence of anal squamous cell carcinoma (ASCC) is rapidly increasing in many countries globally.^1^, ^2^, ^3^ The majority of patients present with localised disease where the primary treatment is chemoradiotherapy (CRT). Six published phase III randomised controlled trials provide much of the evidence supporting this approach.^4^, ^5^, ^6^, ^7^, ^8^, ^9^ However, each trial reported different primary outcomes, and even when the same outcomes appeared to be used, definitions of these outcomes varied (Table 1). This limits between-trial comparison and has resulted in different chemotherapy and radiotherapy regimens being recommended internationally.^10^, ^11^, ^12^, ^13^

**Table 1:** Primary and secondary outcomes in six phase III RCTs of chemoradiotherapy in patients with anal cancer.

Trial (year of publication)	Local treatment failure	Progression-free survival	Disease-free survival	Colostomy-free survival	Colostomy	Acute toxicity	Overall survival	Cancer-specific survival	Local/Regional control
ACT I (1996)	P^1^			S			S	S	
EORTC (1997)	P^1^				S	S	S		
RTOG 87–04 (1996)	P^2^		S	S	S	S	S		S
RTOG 98–11 (2008)			P		S	S	S		S
ACCORD-03 (2012)				P			S	S	S
ACT II (2013)	P^3^	P		S		P	S	S	S

P, primary outcome; S, secondary outcome.

Definition of local treatment failure: 1, clinically, at 6 weeks; 2, on biopsy, post-irradiation; 3, clinically, at 26 weeks.

A core outcome set (COS) is an agreed, standardised set of outcomes that should be measured and reported, as a minimum, in all trials in a specific clinical area. COS are increasingly recognised by research funding bodies, regulators, and journal editors as a means to address outcome heterogeneity and reduce reporting bias. The European Medicines Agency recommends COS use for clinical trials in asthma and the UK National Institute for Health Research (NIHR) recommends outcomes from established COS are included in any new trial proposal.^14^, ^15^, ^16^, ^17^ We previously reported the Core Outcome Research Measures in Anal Cancer (CORMAC) project, an internationally ratified COS for trials of CRT for ASCC, developed through a consensus study involving 149 patient and healthcare professional participants from 11 different countries. The output from CORMAC-COS included 19 outcomes across 4 domains of disease activity, survival, toxicity, and life impact (Table 2).^18^

**Table 2:** CORMAC core outcomes versus PLATO planned outcomes.

	Outcome domain	CORMAC core outcome	Planned PLATO outcome
1	Disease activity	Treatment response	Yes
2		Local failure	Yes
3		Regional failure	Yes
4		Distant failure	Yes
5		Disease progression	No
6		Salvage surgery	Yes
7	Survival	Overall survival	Yes
8		Cancer-specific survival	Yes
9		Disease-free survival	
10		Metastasis-free survival	
11		Progression-free survival	No
12	Toxicity	Anal incontinence	
13		Faecal urgency	
14		Pelvic fistula	
15		Colostomy/ileostomy	Yes
16		Skin loss	
17	Life impact	Physical function	Yes
18		Sexual function	Yes
19		Health related QoL	Yes

Whilst utilisation of the CORMAC-COS will go some way to harmonising outcome reporting in ASCC trials, standardised definitions for each of the outcomes in the COS are required to ensure quality and consistency in measurement and reporting. While COS have been developed for many disease areas, to date very few COS projects have followed through to the necessary next step of recommending standardised outcome definitions. Here we describe the second phase of the CORMAC project (CORMAC-2), in which we established international consensus on standardised definitions of the 11 disease activity and survival outcomes in the CORMAC-COS.

## Methods

### Study overview

CORMAC-2 was conducted through a three step process, initially identifying existing definitions through systematic review; using these to populate a two-round Delphi questionnaire (completed by 51 experts from 13 countries); and finally, ratification through an online consensus meeting. The study protocol was published online a priori.^19^

An international steering committee was established to ensure the validity of the Delphi questionnaire content and promote broader global awareness and participation. Members comprised oncologists, colorectal surgeons, and clinical trialists with leading roles in past and current clinical trials in CRT for anal cancer. The steering committee was formed by e-mail invitation to senior authors of published and active trials CRT for ASCC.

### Patient and public involvement

**Fig. 1:**
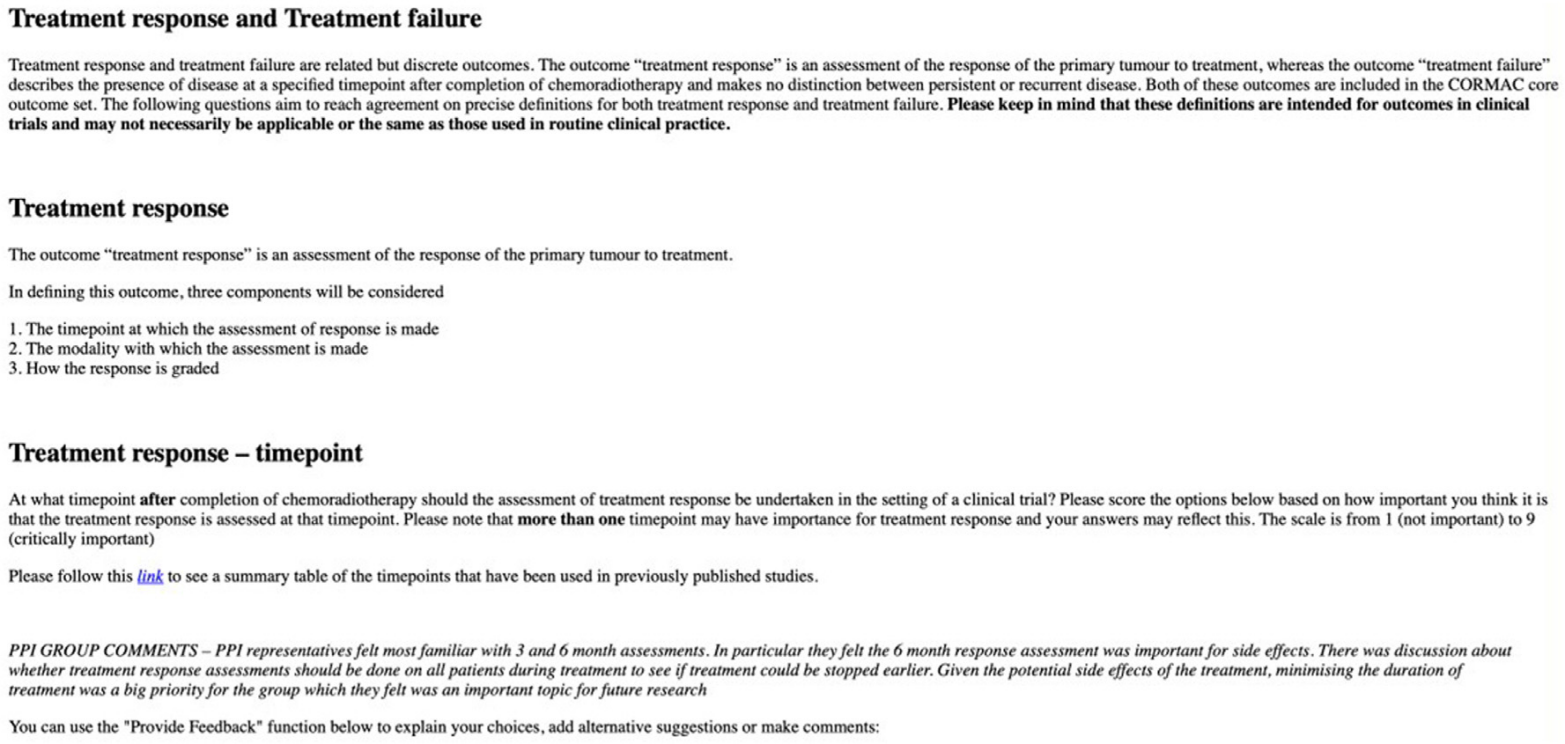
**Screenshot of the Delphi questionnaire, showing a link to further reading and summarised PPI group comments**.

A group of patient and public representatives were recruited through the Leeds Radiotherapy Research Group Public and Patient Involvement (PPI) group (UK) and the Anal Cancer Foundation (USA). The academic research language used to describe nuanced differences in survival and disease activity outcome definitions was considered by the steering committee and the PPI group to be too technical to allow meaningful participation of patients and the public in the Delphi questionnaire and consensus meeting. However, consideration of patient's views was felt to be important especially where outcome definition or measurement may involve burdensome or invasive investigations. PPI groups were therefore asked about the impact and acceptability of different modalities and frequencies of outcome assessment and their feedback was summarised and presented to participants during the Delphi questionnaire (Fig. 1).

### Selection of outcomes

CORMAC-2 focused on outcomes in the disease activity and survival domains, which require a standardised definition. Outcomes in the toxicity and life impact domains require a different approach, involving the identification and recommendation of suitable measurement instruments as described by the COSMIN guidelines.^20^ This represents a substantial piece of work involving different methodology than employed here and is beyond the scope of this phase of the project.

From the survival domain, two outcomes were excluded. Firstly, overall survival (OS) was consistently and unambiguously defined and therefore the steering committee agreed it there was no benefit to including it for voting in the Delphi questionnaire or consensus meeting. Secondly, the identified definitions of progression-free survival (PFS) and disease-free survival (DFS) were found to have significant overlap. After extensive discussion amongst the steering committee, it was decided that due to this overlap, only one would be included and that disease-free survival was the more applicable term in the context of non-metastatic, curative intent trials (the scope of the CORMAC-COS). A summary of the steering committee discussion on DFS versus PFS and the rationale for the decision can be made available from the authors on request.

#### Step 1: systematic review update

The CORMAC systematic review was updated to 11th February 2021. Details of the systematic review, including search strategy, eligibility, and exclusion criteria, can be found on PROSPERO (CRD42016036540). See Appendix 1 for the PRISMA flow diagram. Definitions for the 11 disease activity and survival outcomes in the CORMAC-COS were identified and extracted verbatim. Results from the systematic review update were presented to the steering committee to facilitate accurate summarisation of existing definitions into Delphi question items.

#### Step 2: Delphi questionnaire

##### Recruitment

Healthcare professionals were eligible to participate in the Delphi questionnaire if they have been involved in the design, recruitment, running or publication of anal cancer research and trials. The CORMAC-2 Delphi questionnaire was promoted at the International Multidisciplinary Anal Cancer Consortium (IMACC) conference 2021, through the National Cancer Institute (NCI) Rectal-Anal Taskforce (USA) and active trial networks such as PLATO (PersonaLising Anal cancer radioTherapy dOse, UK) IMACC (International) and subcommittees of the National Cancer Research Institute (NCRI) (UK) and NRG Oncology (North America). Steering committee members used their knowledge of local societies, meetings, email lists and contacts to increase participation in the questionnaire. Potential participants could register their interest via the CORMAC website before the study opened.^21^

##### Questionnaire

Delphi question items were constructed from the outcome definitions identified in the systematic review. Disease activity outcomes were broken down to cover aspects of timing and modality of assessment as well as grading/assessment criteria. Composite outcomes (e.g. disease-free survival) were separated to rate the inclusion of all potential events, as previously described by the Definition for the Assessment of Time-to-event Endpoints in CANcer trials (DATECAN) group.^22^ The Delphi questionnaire was run on the online DelphiManager platform.^23^ Consent to participate was obtained from participants at registration along with demographic information including their discipline, country of practice and role in anal cancer research. During each of the 2 rounds, for each outcome, participants were asked to rate the importance of adopting a particular definition on a Likert scale of 1 (limited importance) to 9 (critical importance). Instructions for completing the questionnaire were included at the start of each round, and links to information necessary for answering questions were provided, for example, details of RECIST criteria.^24^ A summary of the relevant PPI feedback was provided alongside each definition. Participants had the opportunity to provide free text feedback on each question and to suggest alternative definitions (Fig. 1). In round 2, participants were shown the summarised results from round 1, including their own round 1 score for each item, the summarised scores from other participants (as a histogram), and relevant summarised feedback from the free text responses (anonymised). They were then asked to consider this information before re-scoring each item.

##### Consensus criteria

Criteria for consensus were agreed a priori and published in the study protocol. All items from Round 1 were retained for Round 2. After the final round, each definition option was assigned to one of three categories:

1. Consensus in: 70% or more respondents rate the item as critically important (7–9) AND 15% or fewer rate the item as limited importance (1–3). Unless an issue is raised at the consensus meeting, it is included in the final definitions.
2. Consensus out: 50% or less of respondents rate the item as critically important (7–9). Unless an issue is raised at the consensus meeting, it is excluded in the final definitions.
3. No consensus

#### Step 3: consensus meeting

All participants completing both rounds of the Delphi questionnaire were invited to participate in the online consensus meeting along with the members of the steering committee. All participants who registered to take part in the consensus meeting were sent a summary of the Delphi questionnaire results before the meeting. The meeting was chaired by an independent clinician researcher who was not part of the steering committee, had expertise in COS methodology, and in chairing similar meetings (SM). The definitions that met “consensus in” and “consensus out” criteria after the final round of the Delphi questionnaire were presented but were not voted on again unless consensus meeting participants raised a fundamental problem with that definition. “No consensus” definitions were shown, and group discussion was facilitated. The chair ensured different views were heard and all participants could voice their opinions. Following this, anonymous voting was conducted using the same 9-point Likert scale and consensus criteria used in the Delphi questionnaire. If no consensus was found on the first vote, further discussion and a second vote was performed. Anonymous online voting was conducted using Mentimeter software.^25^

### Registration and ethics

The protocol was prospectively registered on protocolexchange.^19^ As per the University of Manchester ethics decision tool, no ethical approval was required as it was a study soliciting professional opinions, all personal information collected was publicly available and participants agreed for their details to be shared as part of collaborative authorship.^26^

CORMAC is registered with the Core Outcome Measures in Effectiveness Trials (COMET) initiative.^27^

### Role of the funding source

There was no funding source for this project.

## Results

### Search strategy and selection criteria

Full details of the systematic review, including search strategy, databases, and selection criteria have been published elsewhere.^28^ Briefly, the systematic review identified 1646 outcomes from 190 trials and observational studies of CRT for ASCC. Outcomes and any accompanying definitions were extracted verbatim from included studies.

### Systematic review & Delphi questionnaire design

Outcome definitions were extracted verbatim and then summarised to allow the identification of similar themes and concepts. For example, within the outcome “treatment failure”, identified concepts included anatomical definition, timing of assessment, modality of assessment and grading of response. For composite time-to-event outcomes, all events were extracted, including definitions of events where provided. The summarised extracted outcomes and concepts were presented to and discussed by the steering committee and the discussion was used to inform the design of the Delphi questionnaire items (Appendix 2 Tables A–F).

For questions about modality of assessment and grading criteria for assessment of treatment failure and treatment response, participants were asked to separately consider small, low-risk tumours (T1-2 ≤4 cm N0 or Nx anal canal or T2 ≤4 cm N0 or Nx anal margin) and large, high-risk tumours (T2 N1-3 or T3-4 N- any). This was because the steering committee felt it likely that different modalities may be preferred based on risk stratification. The criteria used to distinguish these two groups were based on the PLATO trial protocol but it was emphasised to participants that this was just one example of risk stratification and that other definitions may be in use or adopted in future.^29^ The round 1 questionnaire contained 67 options under 15 stem questions covering components of the definition and assessment of 9 outcomes.

### Delphi questionnaire results

50 participants from 13 countries and 7 different healthcare disciplines took part in both rounds of the Delphi questionnaire (Table 3). As a result of participant suggestions after round 1, an additional option was added to the modality of assessment of treatment failure in round 2 (high-resolution anoscopy). Participants in round 1 also fed back that the wording and concepts describing the anatomical definition of local and regional failure were unclear, particularly relating to bony involvement. The steering committee agreed that this would therefore be discussed at the consensus meeting regardless of Round 2 results. After both rounds of the Delphi questionnaire, 36 items met the consensus-in criteria, 24 consensus-out, and 9 reached no consensus. Overall 10 out of the 15 questions reached agreement on all components and 5 questions had components reaching no consensus (Table 4). The attrition between Round 1 and 2 was 34%.

**Table 3:** Round 2 participants by country and profession.

Country	Number	Profession	Number
Australia	1	Clinical Oncologist	15
Canada	1	Colorectal Surgeon	12
Germany	1	Medical Oncologist	6
Italy	1	Radiation Oncologist	12
Netherlands	2	Radiographer	1
Norway	1	Radiologist	3
Poland	1	Radiophysicist	1
Portugal	1		
Spain	1		
Sweden	3		
United Kingdom (UK)	29		
United States of America (USA)	7		
Uruguay	1

**Table 4:** Round 1 questionnaire with Results after both round of Delphi questionnaire and consensus meeting.




EUA, examination under anaesthesia; MRI, magnetic resonance imaging; CT, computer tomography; PET-CT, positron emission tomography; HPV, human papilloma virus.

Green, consensus to include; Red, consensus to exclude; Amber, no consensus.

### Consensus meeting

12 participants from 4 different healthcare disciplines and 7 countries attended the online consensus meeting to ratify the options that had reached consensus-in or consensus-out through the Delphi questionnaire, and to discuss and vote on the options that had not reached consensus. All participants had completed both rounds of the Delphi questionnaire. Clarification of some of the options that had reached consensus-in or consensus-out after the Delphi questionnaire took place but no fundamental problems were raised, and all these items were ratified. Discussions regarding anatomy, bony involvement and radiation were structured using clinical examples to facilitate understanding and clarify definitions. It was accepted that the exemplar clinical scenarios used may be rare in clinical practice but necessary to ensure the utility and robustness of the final definitions. After completion of the Delphi questionnaire and consensus meeting, definitions were agreed for 7 outcomes comprising 16 aspects from 41 individually specified definitions (Table 5).

**Table 5:** CORMAC-2 Agreed definitions for disease activity and survival outcomes.

Domain	Outcome	Aspect	Definition
Disease activity	Treatment response	When treatment response is measured	3 months
			6 months
		Modalities used to assess treatment response–small, low risk tumours	Clinical examination
			MRI scan
		Modalities used to assess treatment response–large, high risk tumours	Clinical examination
			MRI scan
		Criteria used to assess treatment response	Clinical examination and imaging combination assessment: Complete response, partial response, residual thickening, no response.
			Tumour Regression Grading (TRG) system for MRI imaging
	Treatment failure	When treatment failure can be assessed	6 months
		Anatomical locations are included in local failure	Primary tumour site within the anorectum
			Primary tumour site including any directly invaded structures e.g. the vagina
		Anatomical locations are included in regional failure	Inguinal lymph nodes
			Mesorectal lymph nodes
			Presacral lymph nodes
			Internal iliac lymph nodes
			External iliac lymph nodes
			Common iliac lymph nodes
			And/or any disease within the pelvis up to the level of the sacral promontory excluding the bones of the pelvis
		Anatomical locations are included in distant failure	Any tumour deposits outside the pelvis
			Any tumour deposits within the pelvis, including the bones of the pelvis, that are not nodal or primary tumour site
		Additional anatomical information on treatment failure	Definition of local or regional failure includes information on whether the site of failure is inside the radiation clinical target volume (CTV)
		Modalities used to assess treatment failure–small, low risk tumours	Clinical Examination
			Biopsy
			MRI scan
		Modalities used to assess treatment failure–large, high-risk tumours	Clinical Examination
			Biopsy
			MRI scan
			CT scan
			PET-CT scan
	Salvage Surgery	Procedures included in the definition of salvage surgery	Any procedure to excise recurrent/residual tumour following primary chemoratiotherapy. Including but not limited to abdomino-perineal resection; pelvic exenteration and lymphadenectomy
Survival	Overall survival	Events in overall survival	Death due to any cause
	Anal-cancer specific survival	Events in anal-cancer specific survival	Death due to anal cancer Death due to any cause when anal cancer is present
			Death due to treatment for anal cancer
	Disease-free survival	Events in disease-free survival	Local failure
			Regional failure
			Distant failure
			Death due to any cause
	Metastasis-free survival	Event in metastasis-free survival	Distant failure
			Death due to any cause

EUA, examination under anaesthesia; MR, magnetic resonance; CT, computer tomography; PET-CT, positron emission tomography.

### Agreed outcome definitions

Each outcome definition is given below. Where relevant, the nuances from discussion at the consensus meeting are given to explain the decision-making fully and transparently.

#### Treatment response

The outcome “treatment response” is an assessment of the response of the primary tumour and involved lymph to treatment. In defining this outcome, three components were considered: The timepoint at which the assessment of response is made, the modality with which the assessment is made and how the response is graded.

##### Timepoint

Treatment response assessment should take place at 3 months and 6 months after completion of CRT.

##### Modality of assessment (for small, low-risk tumours and large, high-risk tumours)

1. Clinical Examination (patient awake)
2. MRI scan

Both clinical examination with the patient awake and an MRI scan should be used to assess treatment response. It was agreed that a CT scan was not chosen for the modality of assessment (for both small, low-risk and large, high-risk tumours) on the assumption that an MRI scan is performed. If an MRI scan is not available, a CT scan with contrast should be used instead.

##### Assessment criteria/grading

1. Clinical examination and imaging combination assessment—Categorised as complete response, partial response, residual thickening, no response.
2. Tumour Regression Grading (TRG) system for MRI imaging.

A combination of clinical examination and imaging should be used to classify response into complete response, partial response, residual thickening, or no response. The TRG system for MRI imaging should be used, which categorises response from Grade 1 (complete response with no evidence of tumour and normal appearance of the anus) to Grade 5 (no response of the primary tumour or frank tumour progression).^30^ It was recognised that while TRG for MRI imaging is not widely used, mandating it in a trial setting is easier and important to ensure standardisation and reduce heterogeneity.

#### Treatment failure

The outcome “treatment failure” describes the presence of disease at a specified timepoint after completion of CRT and makes no distinction between persistent or recurrent disease. In defining this outcome, three components were considered: The timepoint at which treatment failure can be defined, the definitions of local, regional, distant and radiation field failure, and the modality by which treatment failure can be defined.

##### Timepoint

Treatment failure should be assessed at 6 months after completion of CRT.

##### Anatomical definitions

Local failure is defined as disease at the primary tumour site within the anorectum including any directly invaded structures e.g. the vagina.

Regional failure is defined as disease involving the inguinal, mesorectal, presacral, internal, external iliac, or common iliac lymph nodes. A soft tissue deposit below the sacral promontory that is not from the primary tumour or nodal is also considered a regional failure. Distant failure is defined as any tumour deposit outside the pelvis, or any bony lesions that do not arise from direct invasion by the primary tumour or a regional node.

It was agreed through discussion and voting at the consensus meeting that direct bony invasion from the primary tumour is a local failure, bony invasion from a node is a regional failure and any bony invasion not arising from the primary tumour or node is a distant failure.

##### Radiation field

It was agreed that in addition to information about the anatomical location as above (local, regional, or distant), treatment failure should include information on whether the failure is within the radiotherapy clinical target volume (CTV).

##### Modality of assessment (for small, low-risk tumours)

1. Clinical Examination (patient awake)
2. Biopsy
3. MRI scan

At the consensus meeting it was clarified that in the context of an ASCC clinical trial, confirmation of treatment failure (for both small, low-risk and large, high-risk tumours) should involve histological evidence of invasive disease, whether this is from a biopsy or a surgical resection specimen. Although it is normal practice to have histological confirmation of treatment failure from a biopsy prior to salvage surgery, this is not mandatory in all situations, and confirmation can come from the surgical resection specimen.

##### Modality of assessment (for large, high-risk tumours)

1. Clinical Examination (patient awake)
2. Biopsy
3. MRI scan
4. CT scan
5. PET-CT scan

It was clarified that contrast enhanced CT imaging is required for the assessment of treatment failure in large, high-risk tumours. If PET-CT is performed and includes a contrast CT component, separate contrast enhanced CT is not required. After discussion, it was agreed that PET-CT for assessment of treatment failure in large, high-risk tumours was recommended but not mandatory. The integrity of a trial would not be impacted if PET-CT was not available.

#### Survival

##### Overall survival

Events in overall survival are death due to any cause. This was not asked in the Delphi questionnaire due to unanimous agreement in the literature and amongst the steering committee.

##### Anal-cancer specific survival

Events in anal-cancer specific survival are death due to anal cancer, death due to any cause when anal cancer is present and death due to treatment for anal cancer.

##### Disease-free survival

Events in disease-free survival are local failure, regional failure, distant failure and death due to any cause.

##### Metastasis-free survival

Events in metastasis free survival are distant failure and death due to any cause.

#### Salvage surgery

Discussion about what constituted salvage surgery concluded with unanimous agreement that salvage surgery is any surgical procedure to excise recurrent or residual tumour following primary CRT, including but not exclusive to abdomino-perineal resection, pelvic exenteration and excision of lymph nodes. It was also agreed that the details of the procedure undertaken as salvage surgery should be specifically reported in trials.

## Discussion

CORMAC-2 builds on the CORMAC core outcome set and provides the first internationally agreed definitions for outcomes relating to disease activity and survival for clinical trials of CRT for the treatment of ASCC. All included definitions were agreed by expert healthcare professionals from thirteen different countries and across the spectrum of disciplines involved in anal cancer care and trials. The definitions were agreed using robust and transparent consensus methods to ensure equal representation from all participants. The aim of any COS is to encourage standardised reporting of outcomes in a particular health area. COS utilisation varies across health domains but even when used, outcome definition differences continue to reduce the capacity for data synthesis.^31^ It is also increasingly recognised that understanding the breakdown of composite endpoints is necessary to interpret trial results accurately and gauge the true benefit of an intervention.^32^ We therefore recommend not only that all future trials evaluating CRT for ASCC use the CORMAC core outcome set, but also adopt the outcome definitions agreed in CORMAC-2.

The use of PET-CT did not reach the threshold for inclusion after the Delphi questionnaire. Although not recommended by 2021 ESMO guidelines, PET-CT is increasingly used as part of response assessment in the UK and Europe, with data showing that combined PET-CT and MRI response assessment can predict subsequent outcomes better than either modality alone.^13^^,^^33^ Outside of a trial, in the USA and Canada PET-CT for treatment response assessment cannot be covered by insurance.^34^ The variation in the availability of PET-CT in routine care may partially explain why it did not reach consensus in the Delphi questionnaire, although lack of standardised criteria for grading PET-CT response is also a recognised barrier to its implementation in trials and routine clinical practice.^33^^,^^35^

The use of MRI reached the threshold for inclusion after the Delphi however there was no consensus in the Delphi on use of the TRG system for MRI reporting and this was subsequently discussed at the consensus meeting. Discussion on the day did acknowledge that TRG system is not yet universally adopted however participants proposed that the CORMAC-2 definitions should represent the “ideal” setting and therefore act to drive improvement in trials. At voting following discussion, the TRG system reached the threshold for consensus and is therefore included.

It is recognised that the outcomes and definitions in CORMAC-2 may not be achievable or as relevant in resource limited settings where the trials need pragmatic outcomes that enhance participation and real-world relevance within the limitations of available resources.^36^ However in some domains there are cost effective modifications, such as determining HPV status through immunohistochemistry.

Some components of definitions that did not reach threshold for inclusion following the Delphi questionnaire and consensus meeting may still be of interest in future trials. For example, secondary HPV cancer such as new diagnosis of HPV-associated head and neck cancer as an event for DFS was discussed at the consensus meeting. Although this was not included after voting, all participants felt that this is an important issue that is currently not well researched or reported and future trialists should consider including secondary HPV-associated cancer as an additional outcome beyond those in the CORMAC COS.^36^

The distinction specified between large, high-risk and small, low-risk tumours within the disease activity outcome definitions reflects current clinical practice and understanding of prognostic factors for ASCC. T-stage and N-stage are the most reliable clinical prognostic factors that stratify current clinical guideline recommendations.^37^ TNM staging is used to stratify patients into different trials as part of the PLATO trial, with escalation or de-escalation of standard CRT doses explored within each cohort and the DECREASE trial is also investigating dose de-escalation for early stage disease.^29^^,^^38^ Risk stratification in future trials will likely evolve as the understanding of anal cancer biology improves.^39^ Future trial stratification may be based on biomarkers, such as HPV status or tumour infiltrating lymphocytes (TILs).^40^ In HPV+ disease, adaptive treatment based on cHPV-DNA monitoring is likely both for escalation and de-escalation trials.

Progression-free survival was included as a core outcome in the CORMAC-COS. At the time, questions in the literature about its validity as marker for improved survival or QOL were noted however as it met consensus criteria on voting it was included in the CORMAC-COS.^28^^,^^41^, ^42^, ^43^, ^44^ After gathering all existing definitions for PFS during the first phase of CORMAC-2, it became clear that the event “disease progression” was frequently included in definitions of PFS, however it was rarely further defined. Where disease progression was defined it was frequently described as treatment failure, which rendered the definitions of DFS and PFS effectively the same. Following careful consideration and discussion by the steering committee, it was decided that PFS was of limited relevance outside the context of trials of palliative interventions or metastatic disease. The scope of the CORMAC-COS is trials of CRT for non-metastatic ASCC with curative intent and therefore the decision was taken not to include PFS in CORMAC-2.

Whilst the difficulty caused by unclear and inconsistent definition of outcomes in cancer trials have been widely reported, there remains very little published guidance or recommendations on standardised outcome definitions, and where is has been produced it has often been without formal consensus.^28^^,^^44^, ^45^, ^46^, ^47^ In response to this issue back in 2012, Bonnetain and colleagues planned a series of projects to define time-to-event (TTE) endpoints in cancer trials (DATECAN). Recommendations were published for TTE endpoints in breast, localised colon, renal, and pancreatic cancer, and for gastrointestinal stromal tumours.^22^^,^^48^, ^49^, ^50^, ^51^ No further work from the group has been proposed.

A systematic review undertaken by the COMET initiative in 2020 found that only one-third of COS published up until 2018 made any recommendation on how outcomes should be defined or measured.^52^ The focus of this review however was on outcomes requiring a measurement instrument (e.g. patient reported outcomes such as physical function) rather than clinical or oncological outcomes requiring a definition. Furthermore, this review showed that even where instruments were recommended, many studies did not meet the recommended standards for identifying and selecting outcome measurement instruments.

The CORMAC project is one of very few COS projects registered on the COMET database that has gone on after establishing COS to complete the crucial next step of agreeing standardised outcome definitions and measurement recommendations. Whilst there are recommendations for best practice in how to identify and select outcome measurement instruments, as yet there is no recommended approach for agreeing outcome definitions.^53^ The methods employed in CORMAC-2 were therefore developed based on the recommended consensus methods used for COS development and the methods proposed by the DATECAN initiative. A-priori publication of the CORMAC-2 protocol, clear and transparent reporting of the methods used in each stage, incorporation of patient and carer views and involvement of a broad pool of global experts minimised the potential for bias and maximised our confidence in the final agreed outcome definitions.

One limitation of the CORMAC project is the restriction to the English language which likely limited participation in countries where English is not widely spoken and contributed to the larger proportion of participants from the UK and USA. Although many of the trials within the scope of the CORMAC COS are conducted in the UK and USA, there are ongoing trials in Germany and France.^54^^,^^55^ Whilst there was representation from all disciplines of healthcare professionals involved in anal cancer care and trials, there was a preponderance of clinical/radiation oncologists. Globally, most anal cancer trials are led by clinical/radiation oncologists. Therefore, apart from low participation from Germany (one participant) and France (none), the participants in CORMAC-2 are arguably representative of the groups most likely to use the CORMAC-COS.

Four steering committee members are investigators in the PLATO trial, which could have influenced the content or wording of the Delphi questionnaire. However, the impact of this was minimised through invitation for alternative definitions from all Delphi participants in round 1 and. given the differences in outcome definitions between the PLATO protocol and CORMAC-2, and dropping PFS from CORMAC-2 despite it being the planned secondary outcome for PLATO, this potential influence seems to have been minimal.

Although Delphi is a well-recognised and utilised consensus methodology in healthcare research, it does have some limitations. The sequential rounds of voting and consensus meeting took a long time to complete. As a result, the time between updating the original COMRAC systematic review and the consensus meeting was substantial. Studies published during this time may have influenced questionnaire design and the agreed outcome definitions. Although new studies with unique terms for outcomes were identified in the updated systematic review (from December 2016 to February 2021), once standardised, all new outcomes could be classified into the original framework and the update did not significantly influence the design of the CORMAC-2 questionnaire.

In Delphi methodology, participants do not engage in direct discussions until the consensus meeting. This contrasts with the nominal group methodology, where brainstorming occurs at the beginning of the process. It is possible that wider discussion of questionnaire design and wording beyond the steering group before the Delphi questionnaire. However, during the first round of the Delphi, participants were given the opportunity to suggest new definitions that had not been included and to give free-text feedback, to ensure maximal inclusion of all potential options before proceeding to subsequent rounds. Additionally, the CORMAC-2 consensus meeting was chaired by experienced facilitators ensuring open discussion and lively debate that explored the nuance of practical application of the outcome definitions and significantly improved clarity on the “no consensus” prior to repeat voting.”

The number of participants at the consensus meeting was relatively small compared to the number of participants in the Delphi questionnaire. Consensus meeting participants had to have completed both rounds of the Delphi questionnaire to ensure that all participants were fully informed and engaged with the complex topics to be discussed. The number of consensus meeting participants was carefully considered to balance meaningful engagement and discussion between participants with adequate representation from a spectrum of healthcare professionals. Care was taken not the allow a small number of consensus meeting participants to overturn the results of a larger consensus from the Delphi. Only outcome definitions had not reached consensus through the Delphi or in need of further clarification were discussed and voted on at the consensus meeting.

CORMAC has been cited in updates and studies in the field of ASCC but has yet to be cited in new trial protocols.^37^^,^^56^, ^57^, ^58^, ^59^ CORMAC-2 addresses the lack of accompanying definitions and outcome measurement recommendations for disease activity and survival, but recommendations for measurement instruments for toxicity and life-impact outcomes are still pending. This is an area of active research in ASCC. Since the original CORMAC-COS was published, the QLQ-ANL27 health-related quality of life questionnaire for anal cancer has now completed final international validation.^60^^,^^61^ The ANCHOR trial, which found that the risk of ASCC was reduced with treatment for high-grade squamous intraepithelial lesions compared to active monitoring in patients living with HIV, developed a validated health-related symptom index which may have relevance to patients undergoing CRT for ASCC.^62^^,^^63^ The next phase of CORMAC will be to complete an evaluation of available instruments and recommend specific measurement instruments.

Utilisation of the CORMAC-COS and CORMAC-2 standardised definitions in future trials could significantly improve the quality and utility of data available to inform clinical care. Incorporating them in PLATO and ECOG-DECREASE clinical trials is planned and will further promote awareness and uptake. Data sharing projects such as atomCAT, which uses distributed learning to compare factors associated with outcomes in anal cancer across international centres, will greatly benefit from standardised outcome definitions.^64^

In conclusion, by agreeing on definitions for outcomes in the domains of disease activity and survival, CORMAC-2 will facilitate greater use of the CORMAC COS, increasing outcome standardisation across trials, increasing the quality of data available for clinical decision-making, and ultimately enhancing patient care.

## Contributors

Robert Samuel–conceptualizaton, data curation, formal analysis, investigation, project administration, writing–original draft, writing–review & editing.

Stephen R Knight–data curation, software, formal analysis, writing–review & ediiting.

Richard Adams–conceptualization, methodology, writing–review & editing.

Prajnan Das–conceptualization, methodology, writing–review & editing.

Jennifer Dorth–conceptualization, methodology, writing–review & editing.

David Finch–data curation, software, formal analysis, writing–review & ediiting.

Marianne G. Guren–conceptualization, methodology, writing–review & editing.

Maria A Hawkins–conceptualization, methodology, writing–review & editing.

Susan Moug–project administration, verification, writing–review & editing.

Lakshmi Rajdev–conceptualization, methodology, writing–review & editing.

David Sebag-Montefiore–conceptualization, methodology, writing–review & editing.

Andrew G Renehan–conceptualizaton, data curation, formal analysis, investigation, methodology, writing–original draft, writing–review & editing, supervison.

Rebecca Fish–conceptualizaton, data curation, formal analysis, investigation, methodology, project administration, writing–original draft, writing–review & editing, supervison.

## Declaration of interests

Maria Hawkins is supported by the NIHR University College London Hospitals Biomedical Research Centre. Andrew Renehan is supported by the NIHR Manchester Biomedical Research Centre (IS-BRC-1215-20007). Marianne G Guren is on the Data Monitoring and Ethics Committee for the PLATO trials, is a member of the Norwegian Colorectal Cancer Group (incl anal cancer), editor of writing committee for the Norwegian treatment guidelines for anal cancer, member of the Faculty of Nordic Anal Cancer Group (NOAC) and International Multidisciplinary Anal Cancer Consortium (IMACC) and co-editor of ESMO Anal cancer treatment guidelines 2021 and is a PI in clinical trials that receive drugs for the trial. Prajnan Das has received consulting fees for the American Society for Radiation Oncology and payment/honoraria from Imedex and Bayer. The authors would like to thank The COMET initiative and the University of Liverpool for the use of the DelphiManager software for the CORMAC-2 project at no cost.

## Appendix A Supplementary data

Appendices

Consortia members

## References

[1] Wilkinson J.R., Morris E.J.A., Downing A. (2014). The rising incidence of anal cancer in England 1990-2010: a population-based study. Colorectal Dis.

[2] Islami F., Ferlay J., Lortet-Tieulent J., Bray F., Jemal A. (2017). International trends in anal cancer incidence rates. Int J Epidemiol.

[3] Anal cancer incidence statistics | Cancer Research UK. https://www.cancerresearchuk.org/health-professional/cancer-statistics/statistics-by-cancer-type/anal-cancer/incidence.

[4] James R.D., Glynne-Jones R., Meadows H.M. (2013). Mitomycin or cisplatin chemoradiation with or without maintenance chemotherapy for treatment of squamous-cell carcinoma of the anus (ACT II): a randomised, phase 3, open-label, 2×2 factorial trial. Lancet Oncol.

[5] Peiffert D., Tournier-Rangeard L., Gérard J.P. (2012). Induction chemotherapy and dose intensification of the radiation boost in locally advanced anal canal carcinoma: final analysis of the randomized UNICANCER ACCORD 03 trial. J Clin Oncol.

[6] Ajani J.A., Winter K.A., Gunderson L.L. (2008). Fluorouracil, mitomycin, and radiotherapy vs fluorouracil, cisplatin, and radiotherapy for carcinoma of the anal canal: a randomized controlled trial. JAMA.

[7] Flam M., John M., Pajak T.F. (1996). Role of mitomycin in combination with fluorouracil and radiotherapy, and of salvage chemoradiation in the definitive nonsurgical treatment of epidermoid carcinoma of the anal canal: results of a phase III randomized intergroup study. J Clin Oncol.

[8] Bartelink H., Roelofsen F., Eschwege F. (1997). Concomitant radiotherapy and chemotherapy is superior to radiotherapy alone in the treatment of locally advanced anal cancer: results of a phase III randomized trial of the European Organization for Research and Treatment of Cancer Radiotherapy and Gastrointestinal Cooperative Groups. J Clin Oncol.

[9] Northover J.M.A., Arnott S.J., Cunningham D. (1996). Epidermoid anal cancer: results from the UKCCCR randomised trial of radiotherapy alone versus radiotherapy, 5-fluorouracil, and mitomycin. Lancet.

[10] Radiotherapy dose fractionation, 4th ed. | The Royal College of Radiologists. https://www.rcr.ac.uk/our-services/all-our-publications/clinical-oncology-publications/radiotherapy-dose-fractionation-fourth-edition/.

[11] NCCN guidelines - anal carcinoma. https://www.nccn.org/guidelines/guidelines-detail?category=1&id=1414.

[12] Moureau-Zabotto L., Vendrely V., Abramowitz L. (2017). Anal cancer: French intergroup clinical practice guidelines for diagnosis, treatment and follow-up (SNFGE, FFCD, GERCOR, UNICANCER, SFCD, SFED, SFRO, SNFCP). Dig Liver Dis.

[13] Rao S., Guren M.G., Khan K. (2021). Anal cancer: ESMO Clinical Practice Guidelines for diagnosis, treatment and follow-up☆. Ann Oncol.

[14] Fish R., Sanders C., Williamson P.R., Renehan A.G. (2017). Core outcome research measures in anal cancer (CORMAC): protocol for systematic review, qualitative interviews and Delphi survey to develop a core outcome set in anal cancer. BMJ Open.

[15] COMET Initiative Plain language summaries. https://comet-initiative.org/Resources/PlainLanguage.

[16] Health Technology Assessment (HTA) programme stage 2 guidance notes (REALMS). https://www.nihr.ac.uk/documents/health-technology-assessment-hta-programme-stage-2-guidance-notes-realms/27817.

[17] Agency E.M. (2015). Committee for Medicinal Products for Human Use (CHMP) Guideline on the clinical investigation of medicinal products for the treatment of asthma. http://www.ema.europa.eu/contact.

[18] Fish R., Sanders C., Adams R. (2018). A core outcome set for clinical trials of chemoradiotherapy interventions for anal cancer (CORMAC): a patient and health-care professional consensus. Lancet Gastroenterol Hepatol.

[19] Samuel R., Sebag-Monteore D., Hawkins M. (2022).

[20] Prinsen C.A.C., Vohra S., Rose M.R. (2016). How to select outcome measurement instruments for outcomes included in a ‘Core Outcome Set’ - a practical guideline. Trials.

[21] CORMAC-2 | CORMAC Study. https://cormacstudy.wordpress.com/cormac-2/.

[22] Cohen R., Vernerey D., Bellera C. (2020). Guidelines for time-to-event end-point definitions in adjuvant randomised trials for patients with localised colon cancer: results of the DATECAN initiative. Eur J Cancer.

[23] CORMAC-2 Delphi. https://delphimanager.liv.ac.uk/CORMAC2/Home/Sorry.

[24] Eisenhauer E.A., Therasse P., Bogaerts J. (2009). New response evaluation criteria in solid tumours: revised RECIST guideline (version 1.1). Eur J Cancer.

[25] Interactive presentation software - Mentimeter. https://www.mentimeter.com/.

[26] University of Manchester ethics decision tool. http://www.training.itservices.manchester.ac.uk/uom/ERM/ethics_decision_tool/story.html.

[27] COMET Initiative A core outcome set for clinical trials of chemoradiotherapy interventions for anal cancer (CORMAC): a patient and health-care professional consensus. https://www.comet-initiative.org/Studies/Details/1271.

[28] Fish R., Sanders C., Ryan N., der Veer S.V., Renehan A.G., Williamson P.R. (2018). Systematic review of outcome measures following chemoradiotherapy for the treatment of anal cancer (CORMAC). Colorectal Dis.

[29] Sebag-Montefiore D., Adams R., Bell S. (2016). The development of an umbrella trial (PLATO) to address radiation therapy dose questions in the locoregional management of squamous cell carcinoma of the anus. Int J Radiat Oncol Biol Phys.

[30] Kochhar R., Renehan A.G., Mullan D., Chakrabarty B., Saunders M.P., Carrington B.M. (2017). The assessment of local response using magnetic resonance imaging at 3- and 6-month post chemoradiotherapy in patients with anal cancer. Eur Radiol.

[31] Hughes K.L., Clarke M., Williamson P.R. (2021). A systematic review finds core outcome set uptake varies widely across different areas of health. J Clin Epidemiol.

[32] Walia A., Tuia J., Prasad V. (2023). Progression-free survival, disease-free survival and other composite end points in oncology: improved reporting is needed. Nat Rev Clin Oncol.

[33] Adusumilli P., Elsayed N., Theophanous S. (2022). Combined PET-CT and MRI for response evaluation in patients with squamous cell anal carcinoma treated with curative-intent chemoradiotherapy. Eur Radiol.

[34] NCCN anal carcinoma 2024 guidelines. https://www.nccn.org/professionals/physician_gls/pdf/anal.pdf.

[35] Mirshahvalad S.A., Mesci A., Murad V. (2023). [18F]-FDG PET in anal canal cancer: a systematic review and meta-analysis. Eur J Nucl Med Mol Imaging.

[36] Pramesh C.S., Badwe R.A., Bhoo-Pathy N. (2022). Priorities for cancer research in low- and middle-income countries: a global perspective. Nat Med.

[37] Theophanous S., Samuel R., Lilley J. (2022). Prognostic factors for patients with anal cancer treated with conformal radiotherapy—a systematic review. BMC Cancer.

[38] EA2182/DECREASE home page - ECOG-ACRIN cancer research group. https://ecog-acrin.org/clinical-trials/ea2182-decrease-anus-cancer/.

[39] Guren M.G., Sebag-Montefiore D., Franco P. (2021). Treatment of squamous cell carcinoma of the anus, unresolved areas and future perspectives for research: perspectives of research needs in anal cancer. Clin Colorectal Cancer.

[40] Gilbert D.C., Serup-Hansen E., Linnemann D. (2016). Tumour-infiltrating lymphocyte scores effectively stratify outcomes over and above p16 post chemo-radiotherapy in anal cancer. Br J Cancer.

[41] Shi Q., Sargent D.J. (2009). Meta-analysis for the evaluation of surrogate endpoints in cancer clinical trials. Int J Clin Oncol.

[42] Booth C.M., Eisenhauer E.A. (2012). Progression-free survival: meaningful or simply measurable?. J Clin Oncol.

[43] Oxnard G.R., Morris M.J., Hodi F.S. (2012). When progressive disease does not mean treatment failure: reconsidering the criteria for progression. J Natl Cancer Inst.

[44] Glynne-Jones R., Adams R., Lopes A., Meadows H. (2017). Clinical endpoints in trials of chemoradiation for patients with anal cancer. Lancet Oncol.

[45] Birgisson H., Wallin U., Holmberg L., Glimelius B. (2011). Survival endpoints in colorectal cancer and the effect of second primary other cancer on disease free survival. BMC Cancer.

[46] Whistance R.N., Forsythe R.O., Mcnair A.G.K. (2013). A systematic review of outcome reporting in colorectal cancer surgery. Colorectal Dis.

[47] Hopkins J.C., Howes N., Chalmers K. (2015). Outcome reporting in bariatric surgery: an in-depth analysis to inform the development of a core outcome set, the BARIACT Study. Obes Rev.

[48] Bonnetain F., Bonsing B., Conroy T. (2014). Guidelines for time-to-event end-point definitions in trials for pancreatic cancer. Results of the DATECAN initiative (Definition for the Assessment of Time-to-event End-points in CANcer trials). Eur J Cancer.

[49] Gourgou-Bourgade S., Cameron D., Poortmans P. (2015). Guidelines for time-to-event end point definitions in breast cancer trials: results of the DATECAN initiative (Definition for the Assessment of Time-to-event Endpoints in CANcer trials)^†^. Ann Oncol.

[50] Bellera C.A., Penel N., Ouali M. (2015). Guidelines for time-to-event endpoint definitions in sarcomas and gastro-intestinal stromal tumors (GIST) trials. Results of the DATECAN initiative (Definition for the Assessment of Time-to-event Endpoints in CANcer trials). Ann Oncol.

[51] Kramar A., Negrier S., Sylvester R. (2015). Guidelines for the definition of time-to-event end points in renal cell cancer clinical trials: results of the DATECAN project. Ann Oncol.

[52] Gorst S.L., Prinsen C.A.C., Salcher-Konrad M., Matvienko-Sikar K., Williamson P.R., Terwee C.B. (2020). Methods used in the selection of instruments for outcomes included in core outcome sets have improved since the publication of the COSMIN/COMET guideline. J Clin Epidemiol.

[53] Prinsen C.A.C., Vohra S., Rose M.R. (2016). How to select outcome measurement instruments for outcomes included in a ‘Core Outcome Set’ - a practical guideline. Trials.

[54] Martin D., Balermpas P., Gollrad J. (2020). Radiance - radiochemotherapy with or without Durvalumab in the treatment of anal squamous cell carcinoma: a randomized multicenter phase II trial. Clin Transl Radiat Oncol.

[55] Kim S., Boustani J., Vernerey D. (2022). Phase II INTERACT-ION study: ezabenlimab (BI 754091) and mDCF (docetaxel, cisplatin, and 5-fluorouracil) followed by chemoradiotherapy in patients with stage III squamous cell anal carcinoma. Front Oncol.

[56] Franco P., Segelov E., Johnsson A. (2022). A machine-learning-based bibliometric analysis of the scientific literature on anal cancer. Cancers.

[57] Neibart S.S., Manne S.L., Jabbour S.K. (2020). Quality of life after radiotherapy for rectal and anal cancer. Curr Colorectal Cancer Rep.

[58] Shakir R., Adams R., Cooper R. (2020). Patterns and predictors of relapse following radical chemoradiation therapy delivered using intensity modulated radiation therapy with a simultaneous integrated boost in anal squamous cell carcinoma. Int J Radiat Oncol Biol Phys.

[59] Savoie M.B., Laffan A., Brickman C. (2019). A multi-disciplinary model of survivorship care following definitive chemoradiation for anal cancer. BMC Cancer.

[60] Sodergren S.C., Johnson C.D., Gilbert A. (2018). Phase I-III development of the EORTC QLQ-ANL27, a health-related quality of life questionnaire for anal cancer. Radiother Oncol.

[61] Sodergren S.C., Johnson C.D., Gilbert A. (2023). International validation of the EORTC QLQ-ANL27, a field study to test the anal cancer-specific health-related quality-of-life questionnaire. Int J Radiat Oncol Biol Phys.

[62] Palefsky J.M., Lee J.Y., Jay N. (2022). Treatment of anal high-grade squamous intraepithelial lesions to prevent anal cancer. N Engl J Med.

[63] Atkinson T.M., Lensing S., Lee J.Y. (2023). Construct validity and responsiveness of a health-related symptom index for persons either treated or monitored for anal high-grade squamous intraepithelial lesions (HSIL): AMC-A01/-A03. Qual Life Res.

[64] Theophanous S., Lønne P.-I., Choudhury A. (2022). Development and validation of prognostic models for anal cancer outcomes using distributed learning: protocol for the international multi-centre atomCAT2 study. Diagn Progn Res.

