# Supplementary material for: International consensus to define outcomes for trials of chemoradiotherapy for anal cancer (CORMAC-2): defining the outcomes from the CORMAC core outcome set
Source: eClinicalMedicine. 2024 Dec 5;78:102939. doi: 10.1016/j.eclinm.2024.102939 (PMC11667046; doi:10.1016/j.eclinm.2024.102939)
Supplement: Appendices [file mmc1.pptx]

## Slide 1
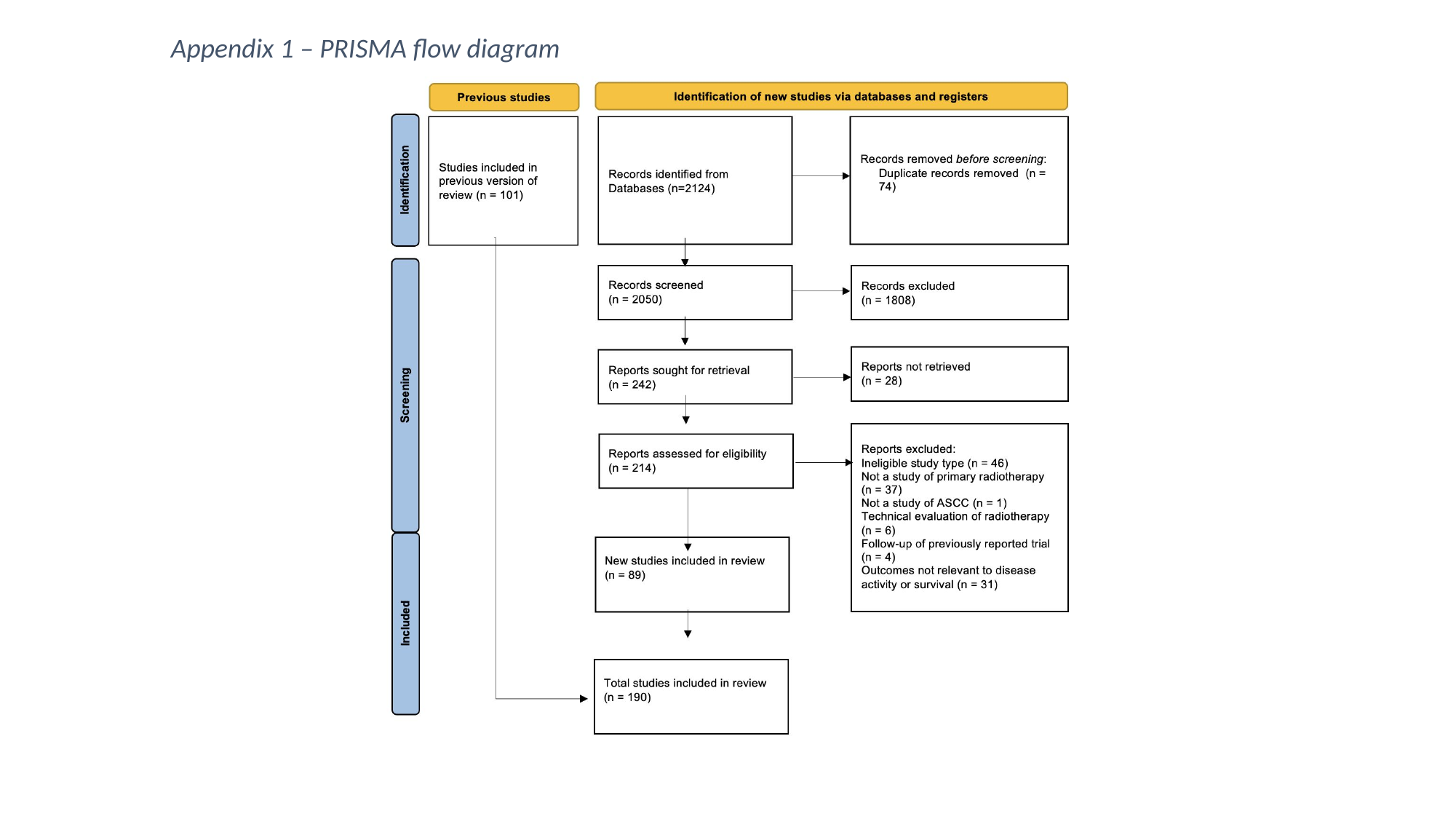

Appendix 1 – PRISMA flow diagram

## Slide 2
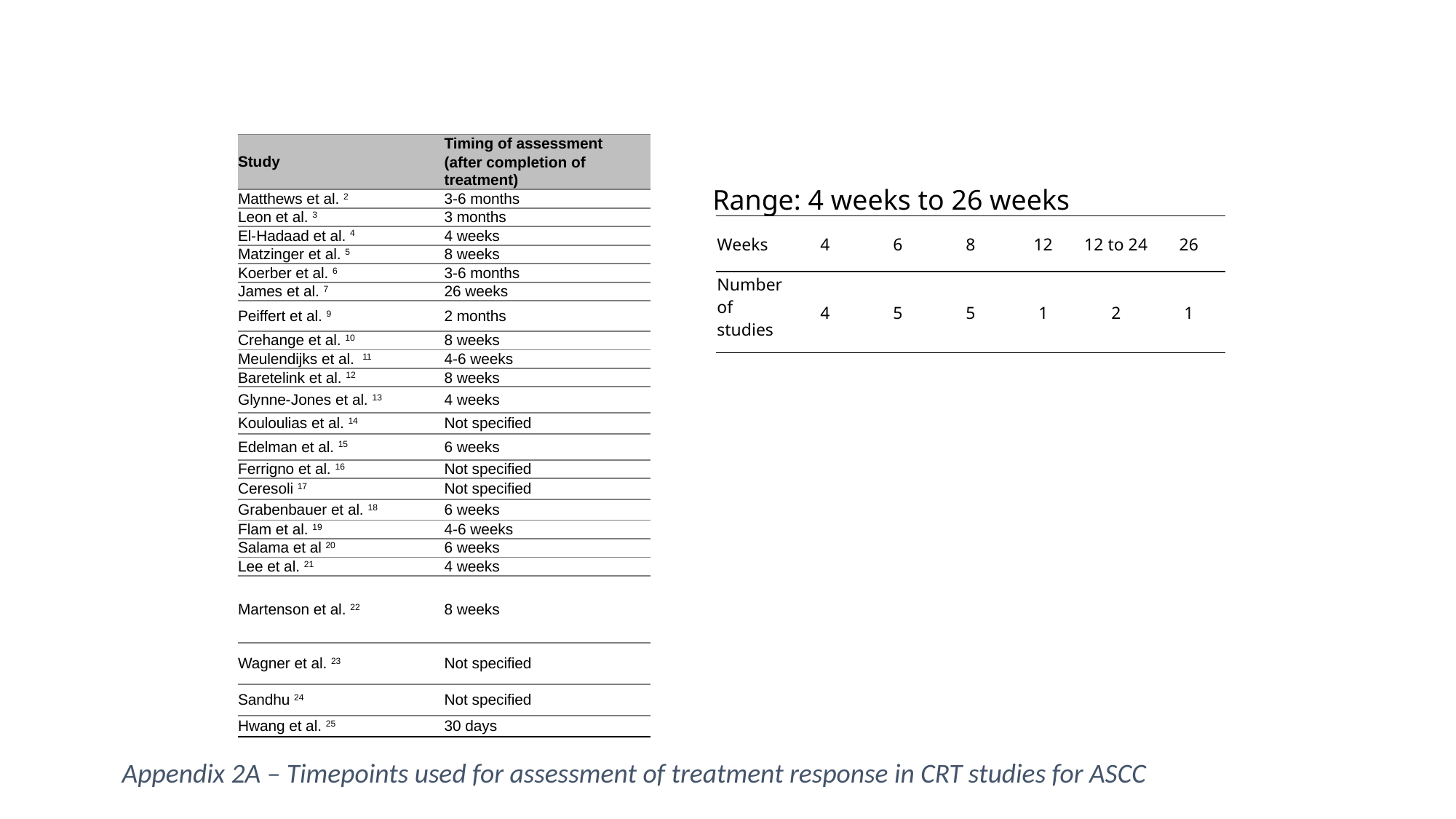

| Study | Timing of assessment |
| --- | --- |
| | (after completion of treatment) |
| Matthews et al. 2 | 3-6 months |
| Leon et al. 3 | 3 months |
| El-Hadaad et al. 4 | 4 weeks |
| Matzinger et al. 5 | 8 weeks |
| Koerber et al. 6 | 3-6 months |
| James et al. 7 | 26 weeks |
| Peiffert et al. 9 | 2 months |
| Crehange et al. 10 | 8 weeks |
| Meulendijks et al. 11 | 4-6 weeks |
| Baretelink et al. 12 | 8 weeks |
| Glynne-Jones et al. 13 | 4 weeks |
| Kouloulias et al. 14 | Not specified |
| Edelman et al. 15 | 6 weeks |
| Ferrigno et al. 16 | Not specified |
| Ceresoli 17 | Not specified |
| Grabenbauer et al. 18 | 6 weeks |
| Flam et al. 19 | 4-6 weeks |
| Salama et al 20 | 6 weeks |
| Lee et al. 21 | 4 weeks |
| Martenson et al. 22 | 8 weeks |
| Wagner et al. 23 | Not specified |
| Sandhu 24 | Not specified |
| Hwang et al. 25 | 30 days |
Range: 4 weeks to 26 weeks
| Weeks | 4 | 6 | 8 | 12 | 12 to 24 | 26 |
| --- | --- | --- | --- | --- | --- | --- |
| Number of studies | 4 | 5 | 5 | 1 | 2 | 1 |
Appendix 2A – Timepoints used for assessment of treatment response in CRT studies for ASCC

## Slide 3
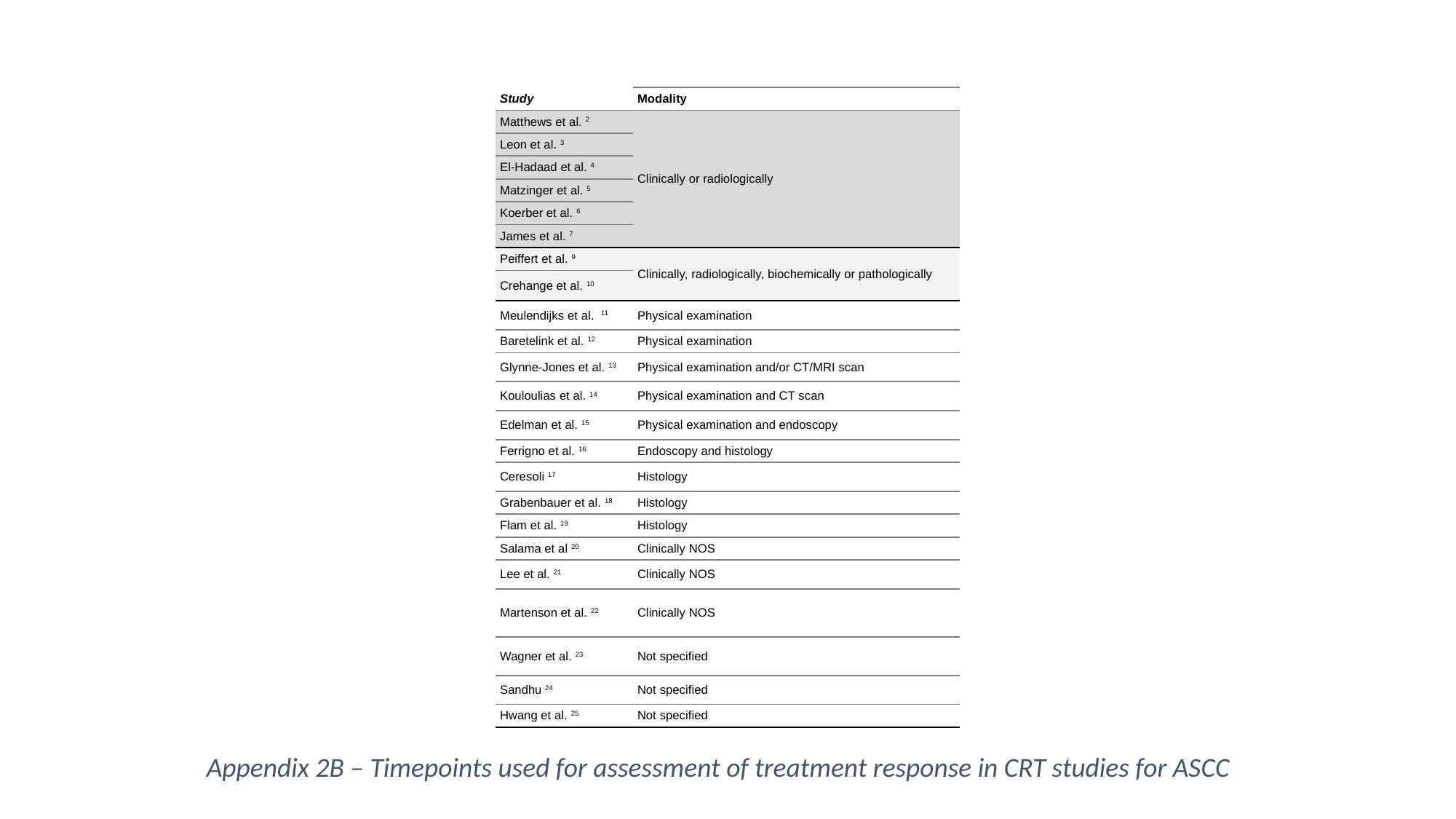

| Study | Modality |
| --- | --- |
| Matthews et al. 2 | Clinically or radiologically |
| Leon et al. 3 | |
| El-Hadaad et al. 4 | |
| Matzinger et al. 5 | |
| Koerber et al. 6 | |
| James et al. 7 | |
| Peiffert et al. 9 | Clinically, radiologically, biochemically or pathologically |
| Crehange et al. 10 | |
| Meulendijks et al. 11 | Physical examination |
| Baretelink et al. 12 | Physical examination |
| Glynne-Jones et al. 13 | Physical examination and/or CT/MRI scan |
| Kouloulias et al. 14 | Physical examination and CT scan |
| Edelman et al. 15 | Physical examination and endoscopy |
| Ferrigno et al. 16 | Endoscopy and histology |
| Ceresoli 17 | Histology |
| Grabenbauer et al. 18 | Histology |
| Flam et al. 19 | Histology |
| Salama et al 20 | Clinically NOS |
| Lee et al. 21 | Clinically NOS |
| Martenson et al. 22 | Clinically NOS |
| Wagner et al. 23 | Not specified |
| Sandhu 24 | Not specified |
| Hwang et al. 25 | Not specified |
Appendix 2B – Timepoints used for assessment of treatment response in CRT studies for ASCC

## Slide 4
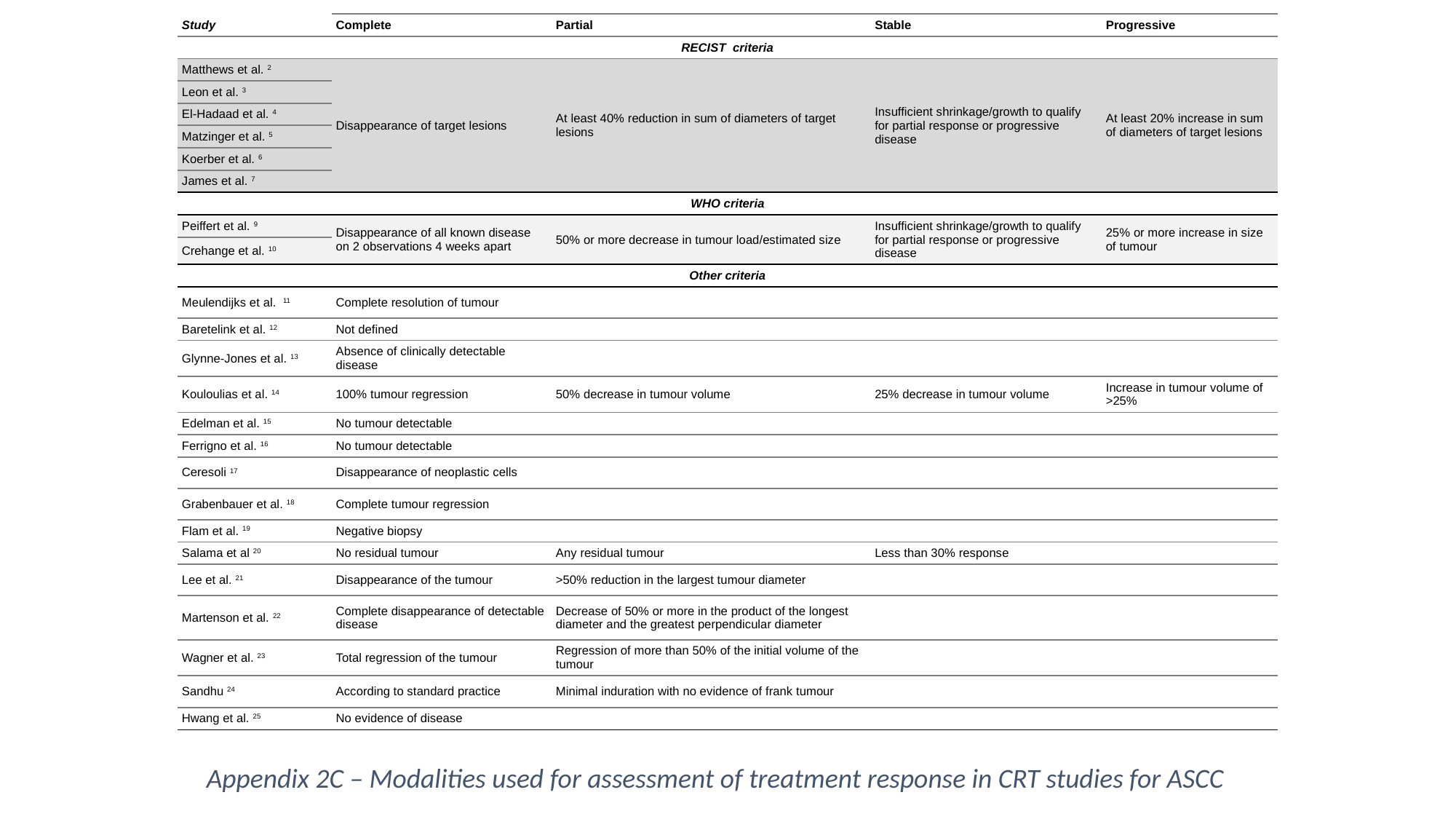

| Study | Complete | Partial | Stable | Progressive |
| --- | --- | --- | --- | --- |
| RECIST criteria | | | | |
| Matthews et al. 2 | Disappearance of target lesions | At least 40% reduction in sum of diameters of target lesions | Insufficient shrinkage/growth to qualify for partial response or progressive disease | At least 20% increase in sum of diameters of target lesions |
| Leon et al. 3 | | | | |
| El-Hadaad et al. 4 | | | | |
| Matzinger et al. 5 | | | | |
| Koerber et al. 6 | | | | |
| James et al. 7 | | | | |
| WHO criteria | | | | |
| Peiffert et al. 9 | Disappearance of all known disease on 2 observations 4 weeks apart | 50% or more decrease in tumour load/estimated size | Insufficient shrinkage/growth to qualify for partial response or progressive disease | 25% or more increase in size of tumour |
| Crehange et al. 10 | | | | |
| Other criteria | | | | |
| Meulendijks et al. 11 | Complete resolution of tumour | | | |
| Baretelink et al. 12 | Not defined | | | |
| Glynne-Jones et al. 13 | Absence of clinically detectable disease | | | |
| Kouloulias et al. 14 | 100% tumour regression | 50% decrease in tumour volume | 25% decrease in tumour volume | Increase in tumour volume of >25% |
| Edelman et al. 15 | No tumour detectable | | | |
| Ferrigno et al. 16 | No tumour detectable | | | |
| Ceresoli 17 | Disappearance of neoplastic cells | | | |
| Grabenbauer et al. 18 | Complete tumour regression | | | |
| Flam et al. 19 | Negative biopsy | | | |
| Salama et al 20 | No residual tumour | Any residual tumour | Less than 30% response | |
| Lee et al. 21 | Disappearance of the tumour | >50% reduction in the largest tumour diameter | | |
| Martenson et al. 22 | Complete disappearance of detectable disease | Decrease of 50% or more in the product of the longest diameter and the greatest perpendicular diameter | | |
| Wagner et al. 23 | Total regression of the tumour | Regression of more than 50% of the initial volume of the tumour | | |
| Sandhu 24 | According to standard practice | Minimal induration with no evidence of frank tumour | | |
| Hwang et al. 25 | No evidence of disease | | | |
Appendix 2C – Modalities used for assessment of treatment response in CRT studies for ASCC

## Slide 5
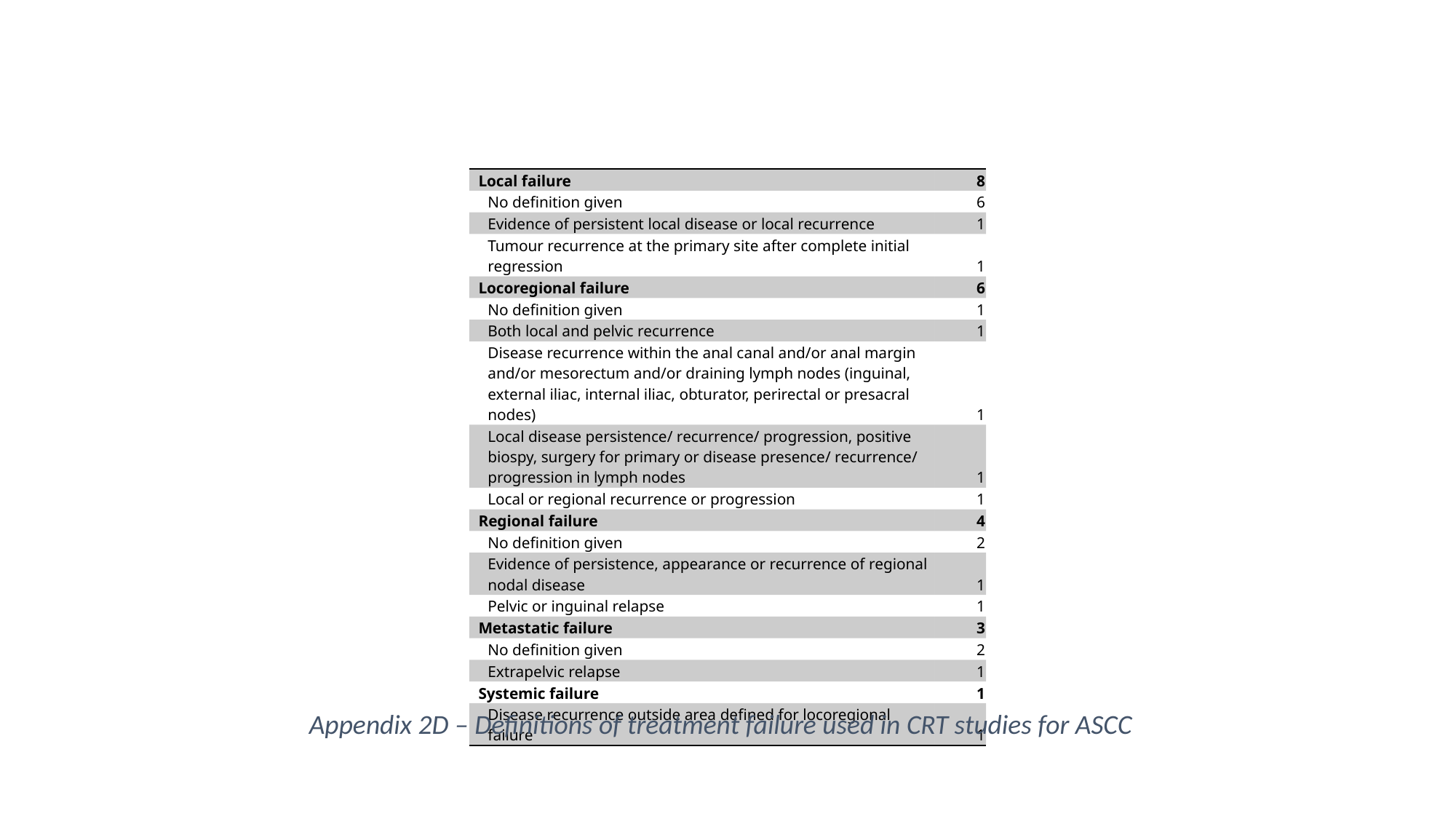

| Local failure | 8 |
| --- | --- |
| No definition given | 6 |
| Evidence of persistent local disease or local recurrence | 1 |
| Tumour recurrence at the primary site after complete initial regression | 1 |
| Locoregional failure | 6 |
| No definition given | 1 |
| Both local and pelvic recurrence | 1 |
| Disease recurrence within the anal canal and/or anal margin and/or mesorectum and/or draining lymph nodes (inguinal, external iliac, internal iliac, obturator, perirectal or presacral nodes) | 1 |
| Local disease persistence/ recurrence/ progression, positive biospy, surgery for primary or disease presence/ recurrence/ progression in lymph nodes | 1 |
| Local or regional recurrence or progression | 1 |
| Regional failure | 4 |
| No definition given | 2 |
| Evidence of persistence, appearance or recurrence of regional nodal disease | 1 |
| Pelvic or inguinal relapse | 1 |
| Metastatic failure | 3 |
| No definition given | 2 |
| Extrapelvic relapse | 1 |
| Systemic failure | 1 |
| Disease recurrence outside area defined for locoregional failure | 1 |
Appendix 2D – Definitions of treatment failure used in CRT studies for ASCC

## Slide 6
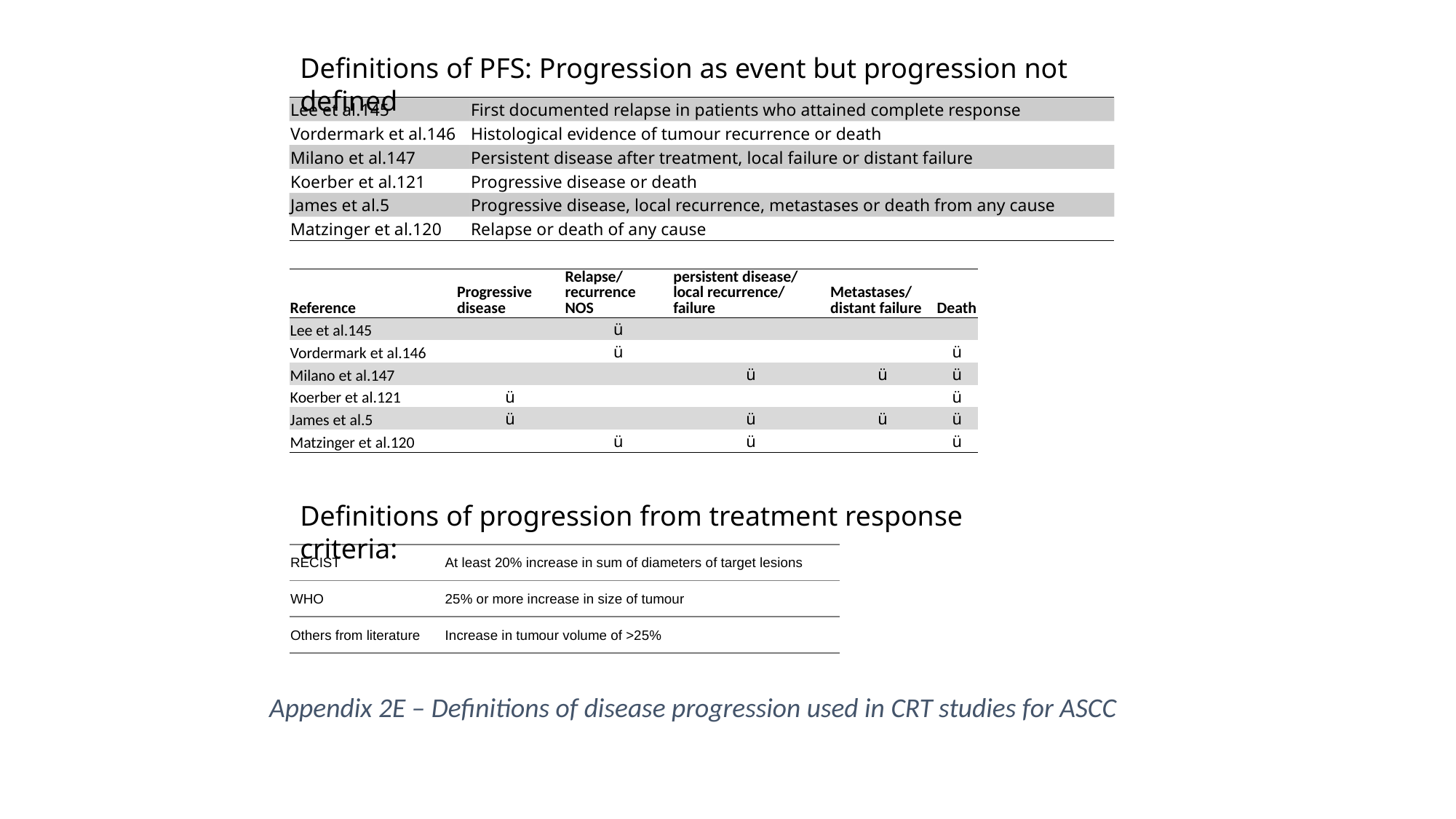

Definitions of PFS: Progression as event but progression not defined
| Lee et al.145 | First documented relapse in patients who attained complete response |
| --- | --- |
| Vordermark et al.146 | Histological evidence of tumour recurrence or death |
| Milano et al.147 | Persistent disease after treatment, local failure or distant failure |
| Koerber et al.121 | Progressive disease or death |
| James et al.5 | Progressive disease, local recurrence, metastases or death from any cause |
| Matzinger et al.120 | Relapse or death of any cause |
| Reference | Progressive disease | Relapse/ recurrence NOS | persistent disease/ local recurrence/ failure | Metastases/ distant failure | Death |
| --- | --- | --- | --- | --- | --- |
| Lee et al.145 | | ü | | | |
| Vordermark et al.146 | | ü | | | ü |
| Milano et al.147 | | | ü | ü | ü |
| Koerber et al.121 | ü | | | | ü |
| James et al.5 | ü | | ü | ü | ü |
| Matzinger et al.120 | | ü | ü | | ü |
Definitions of progression from treatment response criteria:
| RECIST | At least 20% increase in sum of diameters of target lesions |
| --- | --- |
| WHO | 25% or more increase in size of tumour |
| Others from literature | Increase in tumour volume of >25% |
Appendix 2E – Definitions of disease progression used in CRT studies for ASCC

## Slide 7
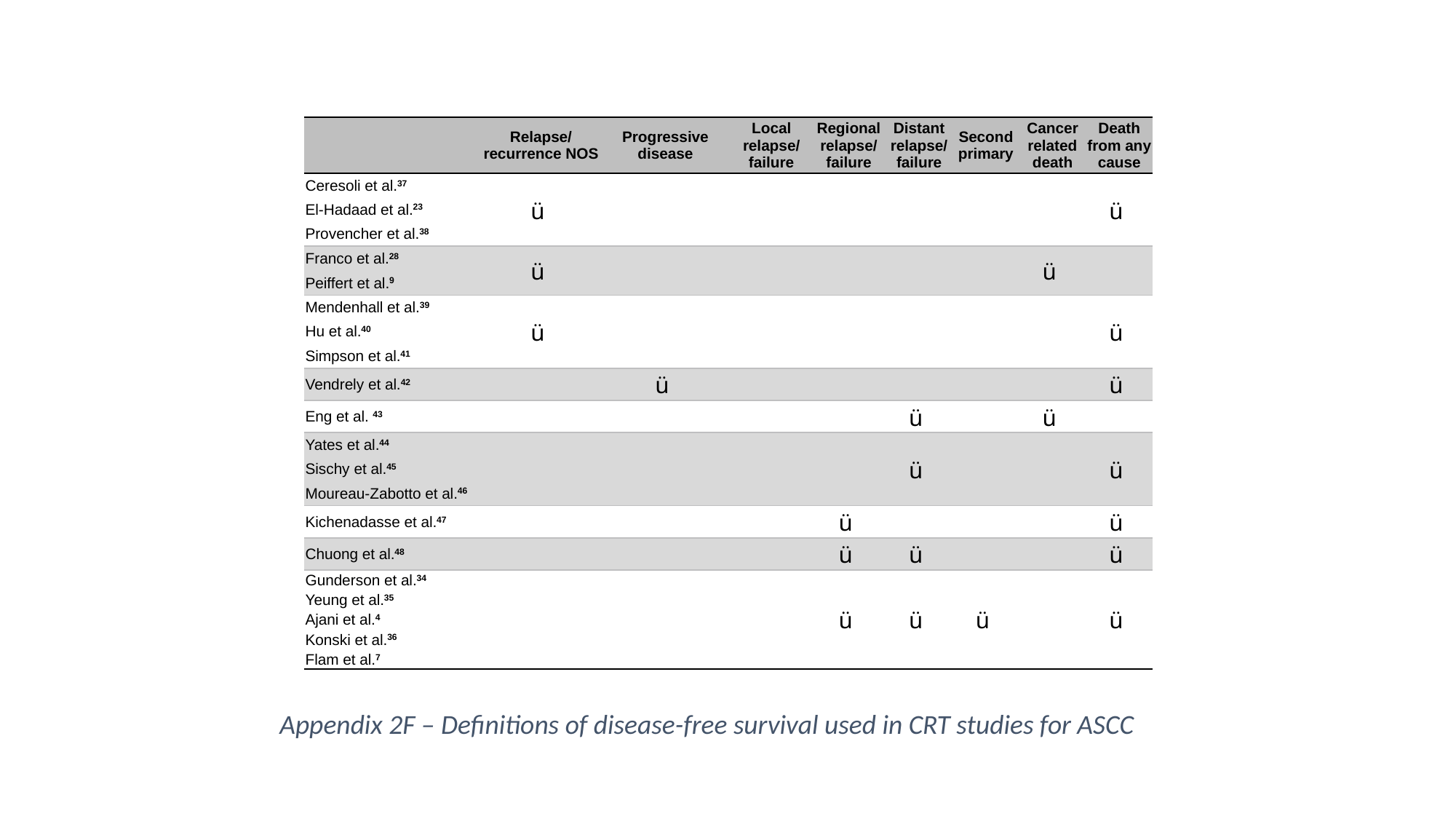

| | Relapse/ recurrence NOS | Progressive disease | Local relapse/ failure | Regional relapse/ failure | Distant relapse/ failure | Second primary | Cancer related death | Death from any cause |
| --- | --- | --- | --- | --- | --- | --- | --- | --- |
| Ceresoli et al.37 | ü | | | | | | | ü |
| El-Hadaad et al.23 | | | | | | | | |
| Provencher et al.38 | | | | | | | | |
| Franco et al.28 | ü | | | | | | ü | |
| Peiffert et al.9 | | | | | | | | |
| Mendenhall et al.39 | ü | | | | | | | ü |
| Hu et al.40 | | | | | | | | |
| Simpson et al.41 | | | | | | | | |
| Vendrely et al.42 | | ü | | | | | | ü |
| Eng et al. 43 | | | | | ü | | ü | |
| Yates et al.44 | | | | | ü | | | ü |
| Sischy et al.45 | | | | | | | | |
| Moureau-Zabotto et al.46 | | | | | | | | |
| Kichenadasse et al.47 | | | | ü | | | | ü |
| Chuong et al.48 | | | | ü | ü | | | ü |
| Gunderson et al.34 | | | | ü | ü | ü | | ü |
| Yeung et al.35 | | | | | | | | |
| Ajani et al.4 | | | | | | | | |
| Konski et al.36 | | | | | | | | |
| Flam et al.7 | | | | | | | | |
Appendix 2F – Definitions of disease-free survival used in CRT studies for ASCC
