# Supplementary material for: International consensus to define outcomes for trials of chemoradiotherapy for anal cancer (CORMAC-2): defining the outcomes from the CORMAC core outcome set
Source: eClinicalMedicine. 2024 Dec 5;78:102939. doi: 10.1016/j.eclinm.2024.102939 (PMC11667046; doi:10.1016/j.eclinm.2024.102939)
Supplement: Consortia members [file mmc2.docx]

# CORMAC-2 collaborators (excluding steering committee members)

| **First Names** | **Last Names** |
| --- | --- |
| Miguel | A. Rodriguez-Bigas |
| Pratik | Adusumilli |
| Ahmed | Allam Mohamed |
| Mario | Alvarez Gallego |
| Eva | Angenete |
| Ane | Appelt |
| Maaike | Berbee |
| Danielle | Brogden |
| Peter | Brown |
| Lucy | Buckley |
| Nathalie | Casanova |
| Rachel | Cooper |
| Nuno | Couto |
| Peter | Coyne |
| Tamzin | Cuming |
| Charlotte | Deijin |
| Kristopher | Dennis |
| Cathy | Eng |
| Alexandra | Gilbert |
| Duncan | Gilbert |
| Karyn | Goodman |
| Rashmi | Jadon |
| Anders | Johnsson |
| Arunansu | Kar |
| Ethan | Ludmir |
| Marie-Louise | Lydrup |
| Ivan | Lyra-Gonzalez |
| Stefania | Manfrida |
| Rebecca | Muirhead |
| Sarah | O'Dwyer |
| Thomas | Rackley |
| Lukasz | Raszewski |
| Leslie | Samuel |
| Mark | Saunders |
| Andrew | Scarsbrook |
| Eva | Segelov |
| Timothy | Simmons |
| Paul | Sutton |
| Nicholas | Symons |
| Deborah | Williamson |
